# Supplementary material for: The psychosocial impact of exercise as an intervention for persons living with obesity and female infertility: A rapid scoping review and pilot study
Source: PLOS Ment Health. 2025 Dec 10;2(12):e0000202. doi: 10.1371/journal.pmen.0000202 (PMC12798248; doi:10.1371/journal.pmen.0000202)
Supplement: S1 Text — Detailed search strategy sample for rapid scoping review, as applied to Medline search. (DOCX) [file pmen.0000202.s001.docx]

**​​S1 Text. Search Strategy for Rapid Scoping Review**

Ovid MEDLINE(R) and Epub Ahead of Print, In-Process, In-Data-Review & Other Non-Indexed Citations and Daily <1946 to December 6th, 2023>

1 exp Obesity/ 265541

2 obes*.tw,kf. 396759

3 1 or 2 450078

4 exp Fertility Agents, Female/ or exp Fertility/ or exp Fertility Clinics/ 67039

5 exp Reproductive Techniques, Assisted/ 80993

6 exp Infertility, Female/ 31254

7 (fertili* or infertili* or fecund* or subfecund* or artificial insemination or embryo transfer or sperm injection or assisted reproduct* or in vitro or IVF or IUI or ICSI or intrauterine insemination).tw,kf. 1698103

8 (reproduct* adj1 (techniqu* or technolog* or assist* or treatment*)).tw,kf. 23991

9 4 or 5 or 6 or 7 or 8 1748196

10 exp "Quality of Life"/ 277975

11 exp Depression/ 153795

12 exp Depressive Disorder/ 123178

13 exp Anxiety/ 114392

14 exp Stress, Psychological/ 154365

15 (Quality of life or Depress* or Anxi* or Mental health or Wellness or Happiness or Stress or Hopeless* or well-being or Wellbeing or HRQL or support* or counsel* or psychosocial).ti,ab. 4010126

16 exp Mental Health/ 64132

17 exp Mental Disorders/ 1453497

18 10 or 11 or 12 or 13 or 14 or 15 or 16 or 17 5081445

19 3 and 9 and 18 3390

20 exp Exercise/ or exp Exercise Movement Techniques/ or exp Exercise Therapy/ 296010

21 exp Sports/ 219324

22 (exercis* or physical fitness or physically fit or walk* or run or running or jog or jogging or swim* or bike or biking or cycling or bicycle or sport or sports).tw,kf. 847303

23 ((weight or resistance or endurance or interval) adj1 train*).tw,kf. 24924

24 (physical activit* or physical condition* or yoga or physically active).tw,kf. 177564

25 weight lift*.tw,kf. 1484

26 20 or 21 or 22 or 23 or 24 or 25 1063543

27 19 and 26 246

28 3 and 9 and 26 749
