# Supplementary material for: The psychosocial impact of exercise as an intervention for persons living with obesity and female infertility: A rapid scoping review and pilot study
Source: PLOS Ment Health. 2025 Dec 10;2(12):e0000202. doi: 10.1371/journal.pmen.0000202 (PMC12798248; doi:10.1371/journal.pmen.0000202)
Supplement: S2 Text — Cochrane risk of bias assessment figure for rapid scoping review [67]. Bias assessment judgments are summarized for articles included in the rapid scoping review through the Cochrane risk of bias assessment tool. Articles 1, 5, and 9 described study protocols only, hence many bias domains were not able to be assessed due to lack of information. (DOCX) [file pmen.0000202.s002.docx]

**S2 Text. Rapid scoping review risk of bias assessment**


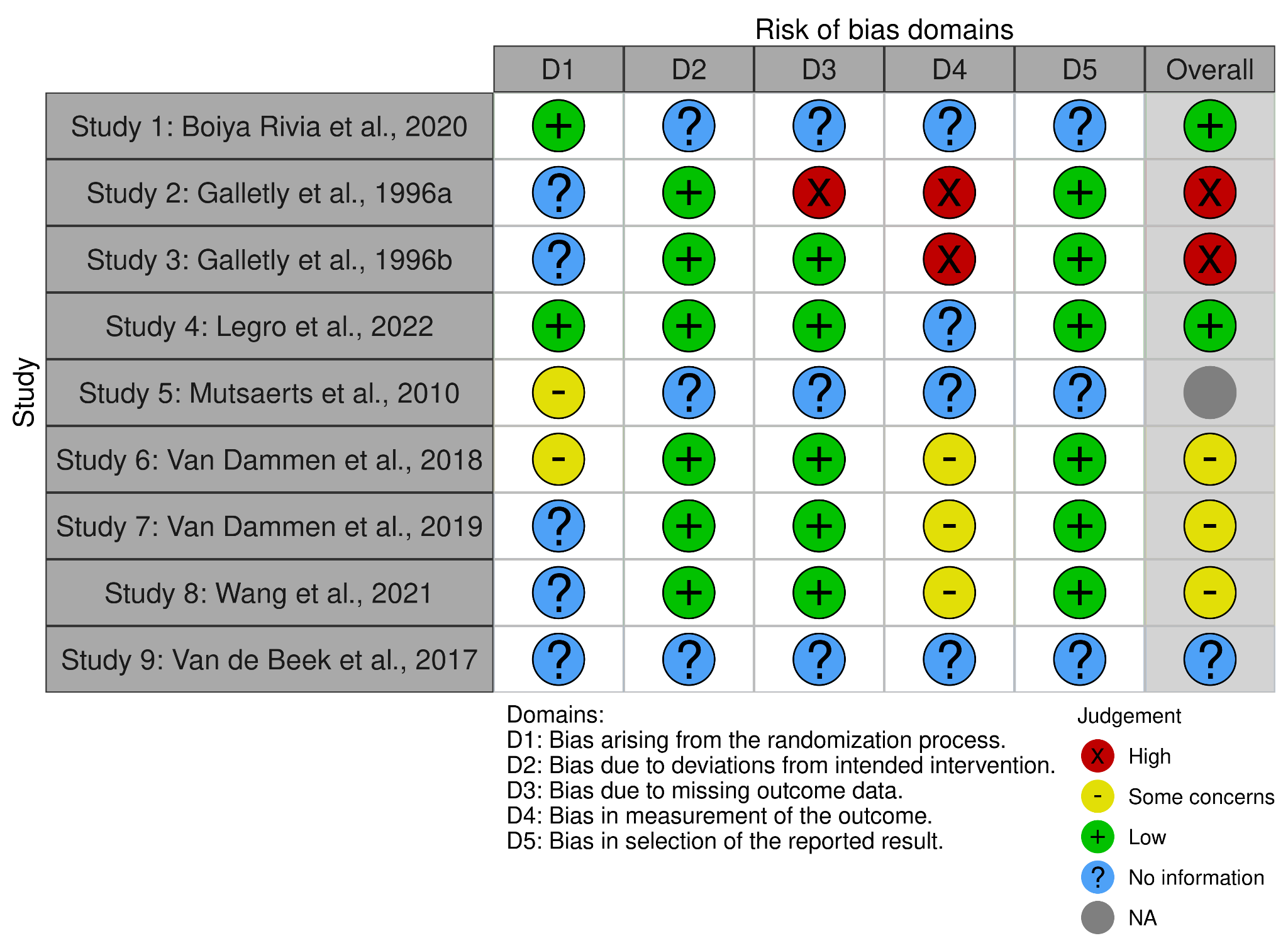


*Legend.* Bias assessment judgments are summarized for articles included in the rapid scoping review through the Cochrane risk of bias assessment tool. Articles 1, 5, and 9 described study protocols only, hence many bias domains were not able to be assessed due to lack of information.
