# Supplementary material for: The psychosocial impact of exercise as an intervention for persons living with obesity and female infertility: A rapid scoping review and pilot study
Source: PLOS Ment Health. 2025 Dec 10;2(12):e0000202. doi: 10.1371/journal.pmen.0000202 (PMC12798248; doi:10.1371/journal.pmen.0000202)
Supplement: Attachment — Submitted filename: Response Letter - PLOS Mental Health.docx [file pmen.0000202.s005.docx]

Katie Wadden, PhD
Memorial University of Newfoundland
September 21, 2024

Academic Editor
*PLOS Mental Health*

Dear Dr. Mabunda Baluku,

We would like to thank you and the reviewers for your thorough evaluation of our manuscript titled “The Psychosocial Impact of Exercise as an Intervention for Persons Living With Obesity and Female Infertility: A Rapid Scoping Review and Pilot Study” (Manuscript ID: PMEN-D-24-00233). We have carefully considered all the comments and suggestions provided by the reviewers and have revised our manuscript accordingly. We believe that the revisions have substantially improved the quality and clarity of our paper.

Below, we provide a detailed point-by-point response to each of the reviewers’ comments. The reviewers' comments are included in bold text, followed by our responses in regular text. The changes made in the manuscript are described where appropriate, with specific page and line numbers indicated.

We hope that our revised manuscript now meets the expectations of the reviewers and the journal, and we look forward to your feedback.

Thank you for your consideration.

Sincerely,

Katie Wadden, PhD
Memorial University of Newfoundland
kwadden@mun.ca

**Reviewer #1**

**Reviewer 1, Comment 1:** This is a nicely written paper on a very interesting topic. An "n" of 7 is just not enough, especially given confounders like the effect of COVID-19. The review is good and would be a nice paper on its own. You should address important topics like a rating of how much the participants enjoyed the exercise. There are many confounding variables which might explain your findings. I strongly encourage you to continue your student and report findings on a larger N in a post-COVID era. The role of Lifestyle Psychiatry in this unique population is a very important topic.

Reviewer 1, Response 1:

1. Thank you very much for your review and for highlighting the novel area of Lifestyle Psychiatry and its potential role in treating persons with obesity and infertility.

We have added the following to our future direction section in the discussion:

Lines 715 to 723: “*The most commonly cited behavioural change counselling approach for persons with infertility and obesity is motivational interviewing (59). Motivational interviewing, which encourages behavioural change by applying a patient-centred counselling style, has been implemented successfully to aid in weight loss before fertility treatment (59). Lifestyle psychiatry, a newer form of behavioral change counselling that promotes exercise for mental health, has shown positive physical and psychological outcomes (60). However, it has not yet been studied in people with infertility and obesity. Integrating lifestyle psychiatry in reproductive medicine could improve both QoL and modifiable risk factors linked to infertility.* ”

2. We agree that the sample size is small, however, we believe that our manuscript uniquely combines data from a pre-experimental pilot study and a rapid scoping review, allowing for a more comprehensive interpretation of our results and the previous study’s findings. By integrating these two approaches, we aimed to enhance the credibility of the pilot study results. To better understand the potential implications of our research in both clinical and academic settings, we have added the following to our limitation section:

Line 686 to 689 : “*The sample size of our pilot study is small. The objective of our pilot study was to perform a small-scale test of our novel methodological approach (e.g., patient-oriented approach and virtual exercise intervention) to inform a larger-scale study (58).* ”

3. Next, we believe it is important not to overlook the value of our novel methodological approach. In developing this pilot study, we collaborated with patient partners who had personal experience with infertility. This collaboration led to a unique, patient-centered approach to the study's design. As a result, beyond the basic findings of the study, our methodological approach holds significant value to the field. It contributes to the existing research by ensuring that the insights from our patient partners directly influenced key aspects of the study, such as the choice of validated questionnaires based on their priorities for an exercise intervention for individuals with obesity and infertility.

To emphasize the significant contribution of our methodology approach to the field, at the beginning of the Methods section we have added the following on Lines 108 - 117:

*“We utilized a dual methodological approach by combining a rapid scoping review and a pilot study to strengthen our findings and patient engagement to ensure the study's objectives and methodology were meaningful to our patient population. Our patient partners shared their experiences, including being diagnosed with infertility, having their BMI measured at the clinic, being referred to an exercise program by their physician, and participating in that program alongside other women with similar infertility experiences. Patient partner feedback helped to determine the selection of validated questionnaires to measure the psychosocial aspects for our pilot study. These measurements had not been included in any studies in our focused review. Thus, implementing patient engagement led to the development of novel study methods and outcomes for a stigmatized population that often lacks a voice (21).”*

4. We had significant challenges recruiting participants due to the COVID-19 pandemic. Furthermore, recruitment in the population is known to be challenging (see Mutsaerts MA, Kuchenbecker WK, Mol BW, Land JA, Hoek A. Dropout is a problem in lifestyle intervention programs for overweight and obese infertile women: a systematic review. Hum Reprod. 2013 Apr;28(4):979-86. doi: 10.1093/humrep/det026. Epub 2013 Feb 20. PMID: 23427235.) Based on powering for pilot studies, the initial sample size for the study was 32 participants. However, many of our participants did not complete the study after signing the consent forms and did not follow up with the research team. Barriers to recruitment are in accordance with previous studies that have documented various challenges with the recruitment and retention of this clinical population for exercise intervention studies. However, staying true to our patient partner’s feedback, who stressed that completing the exercise program as a group would foster a stronger sense of belonging and community among participants, we decided against a rolling start date and had all participants begin the program together. Despite the low enrollment, we chose to continue the study with fewer participants and report our findings descriptively rather than ending the study.

We have added the following to the Limitations Section on Lines 695 to 701:

*“Further limitations included our recruitment strategy. For our pilot study, participants started the exercise intervention at the same time, forming a single cohort. In contrast to a rolling recruitment strategy, where participants begin at different times, our approach ensured our participant cohort experienced social support and the program simultaneously from the outset. However, while this strategy was encouraged by our patient partners, it made recruitment limited as participants were restricted to a certain start and end date.”*

5. Again, comparable to a case study approach, we believe our study adds to the body of literature for this highly under-researched clinical population. Further, the facilitators and barriers experienced by researchers during this pilot study have helped inform further methodological approaches for the larger study that we are currently undertaking and we hope others will take advantage of.

We have now addressed and added the findings from our open-ended questions relating to participants’ satisfaction with the program. Initially, we were unsure if this qualitative piece fit within the manuscript, however, based on your feedback, we have added this.

**Added to the Methods Section, lines 235 to 240:**

*“Patient Satisfaction Questionnaire*

*To gather more information about patient’s satisfaction with exercise intervention, using Google forms, participants responded weekly to open-end questions regarding the program, instructors, how participants felt during the week and any other general comments. Impactful quotes from these responses underwent thematic analysis by two reviewers (CB, KW) and were categorized into subthemes and themes (23).*

*”*

**Added to the Results Section, lines 492 to 510:**

*“Patient Satisfaction Themes*

*Three themes emerged from the open-ended responses on the weekly satisfaction survey that highlighted satisfaction with the program and areas where modifications could be made to enhance participant experience. These themes were: (1) external motivation, (2) taking charge and control of adherence, and (3) the need for resources to overcome barriers. Please see Figure 4, which illustrates the themes and sub-themes for participants’ attitudes towards the exercise intervention.*

*Several participants expressed positive feedback about the instructors and how the instructors motivated them during the intervention. The theme of external motivation emerged as crucial for maintaining adherence to the program, but it also emphasized the potential need to foster intrinsic motivation. The next theme that emerged from the data was taking charge and control of adherence. For example, after periods of missing sessions, participants noted a desire to get back on track with the program and reach their goals. Having adherence goals set, specific session times and a Facebook group likely provided encouragement to continue with the program after setbacks. The last theme was the need for resources to overcome obstacles. There were multiple quotes from participants that identified resources in place to help participants overcome obstacles, such as advice on injuries and providing adequate recovery time but also highlighted where alternative arrangements could be made, such as considering the time of year and weather conditions.*

**Added to the Discussion, lines 630 to 655:**

*“Patient Satisfaction*

*The analysis of open-ended responses revealed three themes about participants' views on the exercise intervention. One theme, "external motivation," highlights how participants saw Kinesiologists as key motivators for adhering to the exercise program. This aligns with other studies, including a systematic review, which found that supervised exercise enhanced motivation and adherence more than unsupervised exercise (54). Interestingly, Hu et al. (2022) compared the effects of online supervised exercise to self-directed exercise in an obese population and showed comparable adherence outcomes (55). Authors concluded that internal motivation may be necessary in an online environment, even in the presence of supervision (56). Building upon discussions of sources of motivation, the second theme was ‘taking charge and control of adherence.’ After noting absent periods, participants documented being motivated to “get back on track” with the study. Further, participants reported adherence-based goals, accountability to ‘live’ session times (e.g., Tuesday and Thursday at 6:00 am), and engagement in the Facebook group were sources of motivation to continue the intervention following setbacks (57). The third theme that merged was the ‘need for resources to overcome barriers.’ Participants highlighted barriers to participation and the desire to overcome these barriers with additional resources. For example, participants discussed that the scheduling of the exercise intervention during the summer months (e.g., taking annual leave during the summer months) and weather conditions (e.g., hot temperatures and lack of air conditioning in the summer months) impacted their ability to adhere to the exercise intervention. In a recent review by Hunter et al. (2021) of randomised controlled trials that utilised physical activity in weight loss interventions for persons living with obesity and experiencing infertility, additional barriers to exercise adherence reported were related to the safety of the neighbourhood and long working hours (56). For persons with a diagnosis of infertility, supervised exercise, sources of motivation and barriers related to logistics, resources and societal factors must be considered when developing and implementing an exercise intervention for this population.”*

Figure 4.

**Reviewer #2**

**Reviewer 2, Comment 1:** The background provides a clear and structured overview of the problem, linking infertility, obesity, and the psychosocial challenges involved. However, the flow could be improved to ensure smooth transitions between ideas.

Reviewer 2, Response 1: Thank you for your feedback on our manuscript. We have made the necessary edits to the background section to provide smoother transitions, more transitional phrases, and shorter sentences as you mentioned. For example, please see the Introduction section, Lines 70 to 74 and 81 to 84:

Line 70 to 74: *“Due to the connection between excess adiposity and dysfunction in processes related to fertility, patients with obesity are often counselled by healthcare providers to reduce their body weight (14). However, there is limited research that examines the psychosocial impacts on persons with obesity and infertility who have been prescribed lifestyle interventions (15)..”*

Lines 81-84: “*In discussion groups with patient partners on our research team, their lived experiences reflected these findings. Patients who did not meet their weight-loss goals reported feeling fear and avoidance toward their healthcare providers at the fertility clinic (18)*.”

**Reviewer 2, Comment 2:** The paragraphs are long and dense, making it difficult to follow the narrative. Breaking them into shorter, more focused paragraphs would improve readability and comprehension.

Reviewer 2, Response 2: We have made the necessary edits to the background section to provide smoother transitions, more transitional phrases, and shorter sentences as you mentioned. For example, please see the introduction section, lines 59 to 68:

*“Women experiencing infertility are more likely to experience higher levels of anxiety (7), depression (8), and psychological distress (9) compared to women not experiencing infertility. Furthermore, there is evidence to suggest that increased stress could negatively impact unassisted conception rates and fertility treatment outcomes (10,11,12). Unfortunately, it appears the relationship between reproductive health and psychosocial factors is bidirectional, meaning these factors often increase and further impact fertility following an infertility diagnosis (7,8,9,10). The severity of infertility is emphasised in a study where women with infertility reported anxiety and depression levels similar to those of women with cancer, heart attacks, and HIV (13). Thus, there is an important need to measure and monitor the psychological well-being of patients being treated for infertility.”*

**Reviewer 2, Comment 3:** There is a lack of smooth transitions between different sections and ideas. Adding transitional phrases or sentences would help guide the reader through the text more effectively.

Reviewer 2, Response 3: We have significantly improved the introduction, methods and discussion sections. For example, please see the discussion section, Lines 89 to 98:

*“The primary objective of the present study is to determine the psychosocial effect of exercise as an intervention, or a component of an intervention, for persons of reproductive age (ages 18-40) living with obesity (BMI > or equal to 28 kg/m^2) and female infertility. To do this, we chose to combine a rapid scoping review with a pilot study as a strategic approach to effectively explore an under-researched area (20). The rapid review aimed to identify gaps in the literature to inform our research objectives and our pilot study focused on exploring psychosocial measures and identifying challenges in implementing an exercise-based intervention for individuals with obesity and infertility, providing real-world context to theoretical insights. This approach improves the validity of findings from a small sample size while addressing unmet needs in the literature.”*

**Reviewer 2, Comment 4:** The dual approach (rapid scoping review and pre-experimental study) is a strength of the study design. Ensure that the rationale for choosing these methods is clear to the reader.

Reviewer 2, Response 4: We have added additional language to the methods section on Lines 108-110 that explain the importance of the dual approach in our methodology.

*“We utilized a dual methodological approach by combining a rapid scoping review and a pilot study to strengthen our findings and patient engagement to ensure the study's objectives and methodology were meaningful to our patient population”*

**Reviewer 2, Comment 5:** The specific measures to be assessed (anxiety, depression, hopelessness, infertility-related stress, and perceived social support) are relevant and comprehensive. It might be helpful to briefly mention why these particular measures were chosen.

Reviewer 2, Response 5: **On lines 162 to 165**, we have added the following language to help clarify why these variables/outcome measures were chosen:

*“During discussion group sessions with patient partners, several key emotional and psychological experiences were shared, including anxiety, depression, hopelessness, infertility-related stress, and perceived social support. From these discussions, the researchers selected validated questionnaires to measure the constructs identified by the patients.”.*

**Reviewer 2, Comment 6:** The rapid scoping review did not include a critical appraisal of the articles due to time constraints. This omission could affect the reliability and validity of the findings since the quality of the included studies is not assessed.

Reviewer 2, Response 6: Based on the criteria for Rapid Scoping Reviews as outlined by Cochrane Training, there is no aspect of critical appraisal for rapid scoping reviews. However, we have added a risk of bias evaluation of each study. The following has been added

**Added to Methods Section, lines 134 to 140:**

***“****JM and KW completed the Cochrane Risk of Bias assessment of each study using need to the following five domains: (1) bias arising from the randomization process; (2) bias due to deviations from intended intervention; (3) bias due to missing outcome data; (4) bias in the measurement of the outcome; (5) bias in the selection of the reported result. Judgement of each study was based on the following rating system: high, some concerns, low and no information. KW acted as the second reviewer and verified all judgments*.*”*

**Added to Results Section, lines 325 to 334:**

*“Risk of Bias*

*The table in Appendix 1. summarises the risk of bias assessment outcomes for studies included in this rapid review. Two studies had a “high” risk of bias due to self-reporting methods and missing outcome data (29,30). Three studies had “some concerns” of bias due to self-reporting methods for outcome measurement (15,31,33). Two studies had a low risk of bias, though both studies were missing information to fully characterise the risk of bias in all domains (26,32). One study had inadequate information to fully characterise the risk of bias (27). Further, articles 1, 5, and 9, describe protocol information only and do not comment on the results of the study (26,27,28). As a result, the risk of bias could not fully be assessed in these studies*.*”*

**Added to the Discussion Section, lines 680 to 682:**

*“Lastly, the articles included in the rapid review had either moderate to high risk of bias or were missing adequate information to assess bias. Because of the risk of bias in included articles, our confidence in the rapid review results is limited.”*

**Reviewer 2, Comment 7:** Both article screening and data extraction were conducted by a single author (JM). This approach could introduce bias and errors in the review process. Involving multiple reviewers in screening and data extraction would enhance the reliability and reduce the potential for individual bias, ensuring a more accurate and comprehensive review.

Reviewer 2, Response 7: Yes, one reviewer deciding which articles should be included introduces a risk of bias. This is why the review is termed as ‘rapid’. Rapid reviews only include one reviewer. This is listed within the limitations section from **Line 675 to 676.**

**Reviewer 2, Comment 8:** The discussion acknowledges deviations in psychosocial outcomes due to methodological variations between the pilot study and those in the rapid review. However, it fails to delve deeply into what specific methodological differences might have caused these variations. More detailed comparison and critical evaluation of methodologies, such as differences in intervention intensity, duration, and participant characteristics, would provide clearer insights into the observed discrepancies.

Reviewer 2, Response 8: Thank you for your feedback. The discussion section has been reviewed to ensure the comments on deviations in psychosocial outcomes between the pilot study and those in the rapid review are more specific. The discussion now highlights methodological differences such as intervention concept influenced psychosocial outcomes This is set out in **Line 543 to 546.**

**“***The studies included in the rapid review included a counseling component to their exercise-based intervention. This difference in intervention concept may have influenced the differences in post-intervention anxiety measures between the pilot study and studies included in the rapid review (29)*.”

**Reviewer 2, Comment 9:** The manuscript starts off strong, but the authors' voice seems to weaken towards the end. The Discussion or Overview section should clearly connect to the study's aim, presenting a broad overview of the results before delving into a more analytical exploration of the implications. Instead, the authors reiterate the studies previously discussed without providing new insights. What is significant about the findings of this review? What should clinicians keep in mind when working with these diagnoses? If additional professional training is necessary, what specific areas do the authors recommend? The lack of a clear takeaway appears to stem from an unclear aim within the manuscript.

Reviewer 2, Response 9: Thank you for your feedback. We agree with the points you have mentioned above. We have made significant changes to the manuscript to connect our study aims, improve the analytical exploration and provide new insight into the field. These changes are highlighted below and have been added to describe the significance of our findings, suggestions for clinicians when working with the study population, and clear takeaways. For example, Please see **lines 708 to 711**, which reinforces our study aims and describes deviations observed across the pilot study and rapid scoping review.:

*“*Results from our rapid review and the inclusion of novel measurements and findings from our thematic and descriptive analyses highlight the need for a critical understanding of how exercise affects our patient population. Furthermore, the unexpected increase in anxiety symptoms and the decrease in QoL in mental health observed in our pilot study suggests the need for continued exploration of the impact of exercise prescription for persons with infertility. ”

Further, please see **lines 713 to 723**, which highlights key takeaways for clinicians:

*“Clinicians should be cautious in expecting exercise to universally enhance mental health outcomes and thus, consider a more holistic approach that includes psychological counselling or other forms of mental health support. For example, the most commonly cited behavioural change counselling approach for persons with infertility and obesity is motivational interviewing (59). Motivational interviewing, which encourages behavioural change by applying a patient-centred counselling style, has been implemented successfully to aid in weight loss before fertility treatment (59). Lifestyle psychiatry, a newer form of behavioral change counselling that promotes exercise for mental health, has shown positive physical and psychological outcomes (60). However, it has not yet been studied in people with infertility and obesity. Integrating lifestyle psychiatry in reproductive medicine could improve both QoL and modifiable risk factors linked to infertility.”*

The **Conclusion Section** reiterates the main aim of the study and implications for practice, lines 736 to 756:

*“Findings from this rapid review and pilot study suggest that while exercise-based interventions can have a positive impact on certain psychosocial outcomes, exercise alone may not fully address the psychological needs of individuals with obesity and infertility. Both rapid review studies and the pilot study measured anxiety, depression, and QoL before and after an exercise-based intervention for women living with obesity and infertility. Findings suggest exercise-based interventions have favorable effects on depression, and physical QoL. Rapid review and pilot study findings suggest exercise-based intervention does not improve mental QoL. This might be explained by infertility-related distress. Rapid review and study findings found neutral to unfavourable effects on anxiety. This effect may be explained by anticipatory anxiety, particularly in rapid review studies. In the pilot study, the COVID-19 pandemic may have introduced unique stressors, unfavourably influencing measures of anxiety. Further, this pilot study adds to the current body of literature by measuring the effects of an exercise-based intervention on hopelessness, perceived social support, and infertility-related stress. Pilot study findings suggest that an exercise-based intervention has favourable effects on hopelessness, perceived social support, and infertility-related stress. To maximize the benefits of exercise programs, evidence from the current study suggests that clinicians should incorporate additional support mechanisms, particularly to manage anxiety and improve mental quality of life. In conclusion, future research should focus on integrating psychological support within exercise interventions and exploring other factors that contribute to the psychosocial well-being of this population.”*

**Reviewer 2, Comment 10:** While the discussion section provides a comprehensive overview of the study's findings and their context within existing literature, it could be improved by addressing the limitations of the study's design and data collection methods more critically.

Reviewer 2, Response 10: We agree with your comments and have made improvements to the limitations section of the discussion on **Lines 656-703**. These improvements include additional explanations around how each limitation or challenge could impact the results of our study. For example, see lines 686 to 690 where we discuss the limitations of our small sample size:

“*The sample size of our pilot study is small. The objective of our pilot study was to perform a small-scale test of our novel methodological approach (e.g., patient-oriented approach and virtual exercise intervention) to inform a larger-scale study (58). The sample size was not sufficiently powered for statistical testing, limiting the analysis to descriptive statistics.*”

**Reviewer #3**

**Reviewer 3, Comment 1: Please provide specific statistical methods**.

Reviewer 3, Response 1: Thank you for your feedback. To improve the specifics of our statistical approach, our methods section from lines 227 to 234 now reads:

*“Data was collected using virtual and physical copies of the questionnaires. Individual scores were inputted into a Microsoft Excel spreadsheet, separated by the questionnaire used and variable measured. Due to the small sample size (n=11), statistical analysis of pilot study data is limited to descriptive statistics. Confidence intervals and P-values for the results are not reliable due to the small sample size of the pilot study (22). A student license was obtained from QualityMetric to analyze the SF-12v2 data. PRO CoRE scoring software was used to score SF-12v2 domains and to analyze means and standard deviations of the physical and mental component scores (PCS, MCS, respectively).”*

**Reviewer 3, Comment 2:** **Please include specific exercise intervention methods**.

Reviewer 3, Response 2: Our exercise intervention has been previously published in the article titled: “PRO-FIT-CARE study: the feasibility assessment of a pilot online exercise intervention for persons living with obesity and female infertility” by Wadden et al., 2024.

We have added the following to the Intervention Section on **Line 205 to 208**:

*“The details of the exercise intervention are outlined in our previous publication, known as the PRO-FIT-CARE study (25). The exercise intervention was designed for participants to exercise at a moderate-to-vigorous intensity level while completing low-impact, body-weighted exercise (25)”*

**Reviewer #4**:

**Reviewer #4, Comment 1:** The objective of the study, as stated in lines 28–29, could be more explicitly formulated. The current phrasing is somewhat fragmented and would benefit from a complete sentence that clearly defines the research objectives. For example, “This study aims to assess the psychosocial impact of exercise interventions on individuals with obesity and infertility through a rapid scoping review and a pilot study.”

Reviewer 4, Response 1: Thank you for your detailed feedback. We have added the suggested language to our abstract on **Line 29**:

“*This study aims to assess the psychosocial impact of exercise interventions on individuals with obesity and infertility through a rapid scoping review and a pilot study”*.

**Reviewer #4, Comment 2:** While the abstract mentions the use of MedLine, Embase, and CINAHL for the rapid scoping review, it does not provide details about the inclusion/exclusion criteria or the specific search terms used. Including a brief mention of these criteria would enhance the transparency of the methodology.

Reviewer 4, Response 2: Thank you for your insight. After reviewing the abstract we agree with your suggestions and have updated the language to reflect our inclusion/exclusion criteria.

**Reviewer #4, Comment 3:** The results section could be more concise and focused. For instance, the presentation of the pilot study findings includes multiple mentions of improvements and declines in different measures, which could be confusing. Grouping the outcomes into broader categories (e.g., improvements in psychological well-being, declines in mental health) could make the results clearer and easier to follow.

Reviewer 4, Response 3: Thank you for your feedback, the results section of the abstract has been updated. Lines 38 to 49 now read:

*“Based on the nine articles from our rapid review, there is evidence that lifestyle interventions combining exercise, diet, and psychobehavioral components improve psychological outcomes like anxiety, depression, quality of life, and self-esteem. In our pilot study, 11 participants (Age: 34 ± 3.7, BMI: 40.3 ± 4.54) engaged in the virtual exercise intervention, and seven completed the post-intervention questionnaires. The results showed mixed psychosocial outcomes. While improvements were seen in depression, hopelessness, physical quality of life, and fertility-related stress, declines were noted in anxiety, mental quality of life, and perceived social support.”*

**Reviewer #4, Comment 4:** The abstract notes an increase in poor mental health following the exercise programme, which contrasts with the general trend observed in the rapid scoping review. However, it does not provide any interpretation or possible explanation for this discrepancy. A brief mention of potential reasons for these findings or the context of the COVID-19 pandemic’s impact could provide valuable context.

Reviewer 4, Response 4: Thank you for the feedback, we have added the following language to **Lines 548 to 554**:

*“According to the WHO, the pandemic resulted in a 25% increase in anxiety and depression globally (43). Levels of depression and anxiety were further increased for patients seeking fertility services during the COVID-19 pandemic (43,4). Fertility patients were affected by clinic closures without any anticipated re-opening date, leading to delayed or cancelled appointments and procedures (4). As a result, the effects of the COVID-19 pandemic and suspensions of fertility treatment may have influenced the mental health of participants in the study, confounding post-intervention measurements of anxiety.”*

**Reviewer #4, Comment 5:** The keywords provided are relevant but could be expanded to include terms like “psychosocial outcomes,” “pilot study,” or “exercise intervention” to improve searchability and alignment with the study’s focus.

Reviewer 4, Response 5: Thank you for your feedback, we have included the terms you mentioned above in the keywords to increase the searchability and alignment with the study’s focus.

**Reviewer #4, Comment 6:** While the background cites relevant literature, it would benefit from a more comprehensive discussion of existing research on the psychosocial effects of infertility and obesity, particularly in the context of prescribed lifestyle interventions. This could help to further emphasize the novelty and importance of the study.

Reviewer 4, Response 6: We have added the following language in lines 59 to 68 to the Background section to discuss the existing research on the psychosocial effects of infertility and obesity:

“*Women experiencing infertility are more likely to experience higher levels of anxiety (7), depression (8), and psychological distress (9) compared to women not experiencing infertility. Furthermore, there is evidence to suggest that increased stress could negatively impact unassisted conception rates and fertility treatment outcomes (10,11,12). Unfortunately, it appears the relationship between reproductive health and psychosocial factors is bidirectional, meaning these factors often increase and further impact fertility following an infertility diagnosis (7,8,9,10). The severity of infertility is emphasised in a study where women with infertility reported anxiety and depression levels similar to those of women with cancer, heart attacks, and HIV (13). Thus, there is an important need to measure and monitor the psychological well-being of patients being treated for infertility.”*

**Reviewer #4, Comment 7:** The transition from discussing infertility and obesity to the introduction of the study's objectives could be smoother. For instance, the discussion of physical activity and its relevance to cardiometabolic health (lines 94–98) feels somewhat abrupt and could be better integrated into the overall narrative. A stronger bridge connecting the general background to the study's specific focus on exercise-based interventions would improve the flow.

Reviewer 4, Response 7: We removed information from aforementioned lines to improve flow from the introduction to the study’s objectives.

**Reviewer #4, Comment 8:** While the background clearly outlines the primary objective, it might benefit from more precise wording regarding the methodology. The term "rapid scoping review" (line 101) is introduced without prior explanation. Clarifying what this entails and why it’s appropriate for the research question would enhance the reader's understanding.

Reviewer 4, Response 8: Thank you for your feedback. We have added information to the background section to better set out the rationale behind choosing a rapid scoping reviewto address our study goals. The following language was added to **lines 91 to 98:**:

*“We chose to combine a rapid scoping review with a pilot study as a strategic approach to effectively explore an under-researched area (20). The rapid review aimed to identify gaps in the literature to inform our research objectives and our pilot study focused on exploring psychosocial measures and identifying challenges in implementing an exercise-based intervention for individuals with obesity and infertility, providing real-world context to theoretical insights. This approach improves the validity of findings from a small sample size while addressing unmet needs in the literature.”*

**Reviewer #4, Comment 9:** The manuscript refers to "persons living with obesity and experiencing female infertility" (lines 103–106). It may be helpful to specify the population's demographic characteristics (e.g., age range, severity of obesity) to give a clearer picture of the study’s focus.

Reviewer 4, Response 9: We have updated the language in the sentence you mentioned above. It now reads:

“*The primary objective of the present study is to determine the psychosocial effect of exercise as an intervention, or component of an intervention, for persons of reproductive age (ages 18-40) living with obesity (BMI > or equal to 28 kg/m^2) and female infertility.*”

**Reviewer #4, Comment 10:** Some sentences could be streamlined to avoid redundancy. For example, the statement about the psychosocial consequences of infertility (lines 74–76) could be more concise, as it is closely followed by another discussion of the psychosocial outcomes targeted by the study (lines 92–93).

Reviewer 4, Response 10: Based on comments from all reviewers, we have heavily edited the introduction to improve flow and reduce redundancy.

**Reviewer #4, Comment 11:** The timeline for the rapid scoping review initiation is unclear (lines 112-113: “June/August 2023”). It is essential to specify the exact start date for clarity. Additionally, the methods for identifying key articles during the initial search (line 118) could be more explicitly detailed, including whether any particular filtering or screening process was used to ensure comprehensiveness.

Reviewer 4, Response 11: We have updated the timeline to be more specific. The rapid review began on June 14th, 2023.

Further, more details have been included pertaining to the screening process for articles included in the rapid review. Please see lines 129 to 140:

*“Articles were screened by one author (JM).* ***Articles identified through the initial search were first screened based on title and abstract. Articles not fulfilling at least one inclusion criteria were excluded at this stage. Next, articles were screened through full text review.*** *Articles were included or excluded using pre-determined inclusion and exclusion criteria, summarized in Table 1.* ***Articles not fulfilling all inclusion criteria were excluded.*** *Due to time constraints, articles were not critically appraised in the present rapid scoping review.* ***Predetermined details from each study were then extracted from each study****. The data extracted from the articles in this review included population data (age, sample size, BMI, and cause of infertility); intervention data; and outcome data (psychosocial outcomes measured and associated results). Data was extracted and charted by one author (JM). Rapid scoping review results are summarized in table format in the results section of this paper.”*

**Reviewer #4, Comment 12:** The decision not to critically appraise articles due to time constraints (lines 123–124) is a limitation of the rapid scoping review. This could be addressed more explicitly by acknowledging the potential impact this might have on the findings. The authors should consider justifying this omission more robustly or suggesting how it could be addressed in future studies.

Reviewer 4, Response 12 Based on comments from previous reviewers and your feedback, we have added explicit language to the limitations section on **Lines 682 to 685**.

**Reviewer #4, Comment 13:**The recruitment process is described in detail, but the reasons for participant dropout (lines 163–167) could be elaborated further. Understanding the challenges of recruiting and retaining participants, especially in a small pilot study, is crucial. This could include a discussion of strategies that could be employed in future research to enhance recruitment and retention.

Reviewer 4, Response 13: We agree with this point. However, our exercise intervention has been previously published in the article titled, “PRO-FIT-CARE study: the feasibility assessment of a pilot online exercise intervention for persons living with obesity and female infertility” by Wadden et al., 2024, which discusses the reason for dropout. We have added this reference to our methods section.

**Reviewer #4, Comment 14:** The criteria for participant selection are clear, but the rationale behind some of the exclusion criteria, particularly regarding metabolic disease and specific infertility diagnoses (lines 173–177), could be expanded upon. Explaining why these particular conditions were excluded would add depth to the methodological rationale.

Reviewer 4, Response 14: We have added the following explanation to the Methods Section on **Line 235** that provides a rationale for our exclusion criteria: “*The exclusion criteria were designed to create a homogeneous study population that allows for clear, interpretable results specific to the effects of an exercise intervention on females living with obesity and anovulatory infertility. They also prioritize participant safety and the validity of the study findings.*”

**Reviewer #4, Comment 15:** While the data collection methods are well documented, the section could benefit from a brief discussion of how missing data (e.g., from participants who did not complete the post-intervention questionnaires) were handled. Additionally, given the small sample size, the limitations of the statistical analysis (limited to descriptive statistics) should be acknowledged more directly, including any potential implications for the interpretation of the results.

Reviewer 4, Response 15: We added the following language to the beginning of the results section starting on **Line 380**:

“*Given the limited sample size, the study's statistical power was insufficient for inferential testing and thus, the reliance on descriptive statistics means that the findings offer preliminary insights. Furthermore, due to the small sample size and dropout rate that could have influenced the results, the generalizability of our findings is limited.*”

Additionally, we added the explanation as to how missing data was handled at the end of the results section on **Line 547**:

*“Data from participants who completed only the pre-intervention questionnaires were included in the analysis of baseline characteristics, providing insight into the baseline psychosocial profiles of those who started the program. Missing data from participants who did not complete the post-intervention questionnaires were not imputed. The absence of post-intervention data from these participants introduces limitations as the full impact of the exercise intervention could not be assessed for those individuals. This incomplete data set significantly impacts the robustness of the study's results. However, it also underscores the challenges of participant retention in exercise-based interventions.”*

**Reviewer #4, Comment 16:** The results section is solid but would benefit from additional analysis and discussion. Enhancing clarity, expanding on the statistical analysis, and interpreting the findings in a broader context would significantly strengthen the manuscript.

Reviewer 4, Response 16: We expanded upon our statistical analysis and have included further discussions on the interpretation of the findings in the discussion section based on comments from reviewers.

**Reviewer #4, Comment 17:**The results section is well organised, presenting the scoping review, study characteristics, and pilot study outcomes in a logical sequence. However, there is a need for clearer transitions between the different parts of the section. Explicitly demarcating the scoping review results and pilot study findings with headings could enhance readability.

Reviewer 4, Response 17: Thank you for your feedback. We have now added headers to the discussion section.

**Reviewer #4, Comment 18:** The rapid scoping review process is described adequately, but the exclusion criteria for full-text screening and reasons for article exclusion should be more detailed to ensure transparency and reproducibility. The PRISMA diagram is referenced but not provided, which limits the ability to assess the thoroughness of the screening process. Including this figure is essential.

Reviewer 4, Response 18: Upon initial submission, we attached the PRISMA diagram as an appendix but it appears that it did not make its way through to the reviewers. We have included it once again in the updated manuscript.

**Reviewer #4, Comment 19:** The summary of the studies in Table 2 is comprehensive but can be overwhelming due to its dense format. Consider splitting the table into multiple parts or providing a more condensed summary focussing on key points. Some studies reported significant outcomes while others did not, but there is no discussion or interpretation of these mixed results. It would be helpful to provide an analysis of why certain studies showed significant outcomes and others did not.

Reviewer 4, Response 19: This is an excellent point. We designed Table 2 to be comprehensive and include all necessary information in one single table for readers to review. We have now split Table 2 into two separate tables. Please see Table 3 which specifically focuses on the outcomes of the studies.

Additionally, in a separate table, we have completed a Risk of Bias assessment on the reviewed articles. This has been added to the results section along with a discussion on the result of this assessment.

We have added a discussion on the mixed results in the discussion section.

**Reviewer #4, Comment 20:** The results of the pilot study are intriguing, but the sample size is small and there is a significant dropout rate. These limitations should be more explicitly acknowledged, as they impact the generalisability and robustness of the findings. The change in BMI post-intervention is mentioned, but the clinical relevance of this reduction is not discussed. Including a discussion on whether this change is meaningful in the context of infertility and obesity would be valuable.

Reviewer 4, Response 20: Currently, a 5 to 10% reduction in body weight has been shown to significantly impact the fertility-related outcome. However, the objective of the Wadden et al. (2024) feasibility manuscript was to discuss weight-centric versus cardiorespiratory approaches to improvements in fertility outcomes. Furthermore, weight loss was not included as a primary outcome or measure of interest for the present study. The focus of our discussion is on the examination of the psychosocial measures before and after an exercise intervention.

**Reviewer #4, Comment 21:** There is a lack of detailed statistical analysis in the results of the pilot study. The manuscript would benefit from a description of the statistical tests used to assess pre- and post-intervention changes, along with p-values or confidence intervals.

Reviewer 4, Response 21: Due to the small sample size (n<30), we feel that p-values and confidence intervals are unreliable for our outcome measures. Please see the following references that support the use of descriptive statistics for pilot studies: Lee, E.C., Whitehead, A.L., Jacques, R.M. *et al.* The statistical interpretation of pilot trials: should significance thresholds be reconsidered?. *BMC Med Res Methodol* 14, 41 (2014). https://doi.org/10.1186/1471-2288-14-41

**Reviewer #4, Comment 22:** The conclusion that exercise-based interventions may improve psychological outcomes in the short term is reasonable, but the absence of long-term effects should be discussed in more depth. The manuscript should explore potential reasons for this decline in effect over time. The mixed results on physical and mental QoL, particularly in the LIFEstyle study, deserve more thorough discussion. The implications of these findings for clinical practice and future research should be considered.

Reviewer 4, Response 22: We have added additional exploration of long-term effects of psychological outcomes using the LIFEstyle study on Lines **724-734**:

*Lastly, understanding the long-term effects of lifestyle interventions, including exercise-based programs, on psychosocial outcomes for individuals with infertility is crucial. In our pilot study, a reduction in self-reported anxiety and mental QoL from baseline suggests that time may significantly impact these outcomes (8). Studies by Van Dammen et al. (2019) and Van de Beek et al. (2017) found that the benefits observed immediately after the intervention diminished over time, with no significant differences in QoL, perceived stress, or sleep quality at five years (15, 28). The lack of sustained improvement in psychosocial measures for individuals with obesity and infertility underscores the need for revising intervention designs to enhance their long-term effectiveness. Overall, future research should focus on integrating psychological support within exercise interventions and exploring other factors that contribute to the long-term psychosocial well-being of this population.*

**Reviewer #4, Comment 23:**

Minor Points:
- Ensure consistent referencing of studies in the text and tables (e.g., "Galletly et al., 1996a" vs. "Galletly et al., 1996b").
- There is a minor typographical error in the sentence on line 227: "searing" should be "searching."

Reviewer 4, Response 23: We have corrected the referencing and typographical errors mentioned above.

**Reviewer #4, Comment 24:** The section on variations in psychosocial outcomes could be strengthened by exploring specific factors like sample characteristics, intervention duration, or measurement tools that might explain these deviations.

Reviewer 4, Response 24: We have added the following language to the discussion section on **Line 547**:

*“Differences in anxiety and depression-related outcomes between the pilot study and studies included in the rapid review may be due to the inclusion of a counseling component to the exercise-based intervention in the studies included in the rapid review. This difference in intervention concept may have influenced the differences in post-intervention anxiety measures between the pilot study and studies included in the rapid review (29). ”*

**Reviewer #4, Comment 25:** Addressing Confounding Factors: The consideration of the COVID-19 pandemic as a confounding factor is commendable and adds validity to the study. To further improve, the discussion could provide more detailed insights into how the pandemic specifically affected the study participants, rather than relying solely on global statistics.

Reviewer 4, Response 25: Thank you for your feedback regarding the addition of insights into how the pandemic affected the study participants. We explained in the discussion and limitations sections how the pandemic impacted our methods and may have impacted our results. Furthermore, we have now included our findings from our open-ended questions related to participants’ satisfaction with the program. These findings provide insight into the challenges faced by our participants during the pandemic.

**Reviewer #4, Comment 26:** The discussion is well-structured, with clear subsections. However, smoother transitions between sections, such as between anxiety, depression, and quality of life, would enhance coherence.

Reviewer 4, Response 26: We have added additional language to the discussion section that allows for a smoother transition from the discussion about anxiety and depression to quality of life.

For example:
“*In addition to anxiety and depression, Quality of life (QoL) was also measured in the study.*”

**Reviewer #4, Comment 27:** The inclusion of unique psychosocial measures, like hopelessness and infertility-related stress, adds depth to the study. The discussion could be improved by providing context on why hopelessness was not included in previous studies and what its inclusion reveals about the studied population.

Reviewer 4, Response 27: We agree with your suggestion of expanding on our inclusion of findings of hopelessness. We have added the following language to **Line 585**:

*“Our patient partners identified the feeling of hopelessness as a significant experience throughout the discussion sessions. Interestingly, only 20% of our participants in the pilot study reported mild hopelessness while the remaining 80% reported levels of hopelessness within the normal range. Future research should explore the role of hopelessness in people experiencing infertility.”*

**Reviewer #4, Comment 28:** The strengths and weaknesses are discussed candidly. However, while the limitations are thoroughly covered, the strengths, particularly the patient-centred approach and integration of patient feedback, could be emphasized more to highlight the study’s significance.

Reviewer 4, Response 28: We have updated the strengths and weaknesses based on feedback from reviewer 2 and we feel as though we have included additional emphasis on the use of a patient-oriented approach.

**Reviewer #4, Comment 29:** The discussion could benefit from a more explicit conclusion that summarises the key findings and their implications, along with specific recommendations for future research or clinical practice, such as incorporating psychological support into exercise-based interventions.

Reviewer 4, Response 29: We have added additional language to the discussion on **Lines 741:**

*“Furthermore, our study offers new insights by examining the effects of exercise on hopelessness, perceived social support, and infertility-related stress, all of which showed favourable outcomes. In conclusion, future research should focus on integrating psychological support within exercise interventions and exploring other factors that contribute to the psychosocial well-being of this population.”*

**Reviewer #4, Comment 30:**

Other Recommendations:
a) Provide a detailed analysis of why certain psychosocial outcomes differed from previous studies.
b) Offer more context on how the COVID-19 pandemic specifically impacted the study participants.
c) Improve transitions between discussion sections for better coherence.
d) Expand on the significance of including hopelessness as a measure in the context of infertility and obesity.
e) Balance the discussion of limitations with a stronger emphasis on the study’s strengths.
f) Conclude with a clear summary of key findings and practical recommendations for future research and interventions.

**Reviewer #4, Comment 31:** The conclusion is clear and effectively summarises the key findings. However, it could benefit from a more structured approach, separating the summary of findings from the implications.

Reviewer 4, Response 31: We have restructured the conclusions to separate the findings from the implications.

**Reviewer #4, Comment 32:** The integration of rapid review and pilot study findings is well done. To strengthen this section, the authors could more explicitly compare and contrast these findings.

Reviewer 4, Response 32: We have added additional language to the discussion section on **Lines 556-566**.

**Reviewer #4, Comment 33:** The conclusion touches on potential explanations for the observed effects, such as infertility-related distress and the impact of COVID-19. Expanding on the implications for future research or practice would enhance this section.

Reviewer 4, Response 33: Thank you for your feedback, we have rewritten the conclusion paragraph based on feedback from Reviewer 2 and have incorporated additional comments about the impact of COVID-19 on the interpretation of our results.

**Reviewer #4, Comment 34:** The conclusion effectively supports the findings but could avoid broad generalisations. For example, the statement on mental quality of life could be softened to reflect context-dependent factors.

Reviewer 4, Response 34: Thank you for your feedback, we have rewritten the conclusion paragraph based on feedback from Reviewer 2 and have softened statements to reflect context-dependent factors.

**Reviewer #4, Comment 35:** Adding a sentence on future research directions or practical implications would provide a forward-looking perspective.

Reviewer 4, Response 35: Based on feedback from Reviewer 2, we have included additional language in the conclusion paragraph about future research directions and practical implications to provide a forward-looking perspective.

**Reviewer #4, Comment 36:** The conclusion could end with a stronger, more impactful statement, emphasising the importance of addressing both physical and psychosocial outcomes in interventions.

Reviewer 4, Response 36: Thank you for your feedback, we have rewritten the conclusion based on feedback from another reviewer and feel as though it now ends with a stronger, more impactful statement that emphasizes the importance of addressing both physical and psychosocial outcomes in interventions.
