# Supplementary material for: The psychosocial impact of exercise as an intervention for persons living with obesity and female infertility: A rapid scoping review and pilot study
Source: PLOS Ment Health. 2025 Dec 10;2(12):e0000202. doi: 10.1371/journal.pmen.0000202 (PMC12798248; doi:10.1371/journal.pmen.0000202)
Supplement: Attachment — Submitted filename: Response to Reviewers - May18_25,PLOSMH.docx [file pmen.0000202.s006.docx]

May 18th, 2025

Staff Editor
*PLOS Mental Health*

Dear Dr. Avanti Dey,

We would like to thank you and the reviewers for your thorough evaluation of our manuscript titled “The Psychosocial Impact of Exercise as an Intervention for Persons Living With Obesity and Female Infertility: A Rapid Scoping Review and Pilot Study” (Manuscript ID: PMEN-D-24-00233). We have carefully considered all the comments and suggestions provided by the reviewers and have revised our manuscript accordingly. We believe that the revisions have substantially improved the quality and clarity of our paper.

Below, we provide a detailed point-by-point response to each of the reviewers’ comments. The reviewers' comments are included in bold text, followed by our responses in regular text. The changes made in the manuscript are described where appropriate, with specific page and line numbers indicated.

We hope that our revised manuscript now meets the expectations of the reviewers and the journal, and we look forward to your feedback.

Thank you for your consideration.

Sincerely,

Katie Wadden

*Memorial University of Newfoundland*

**Comments from Reviewer**

**Reviewer #5:** Although I am new to this manuscript, I can see that the authors have adequately addressed the previous concerns of Reviewers 1 to 4. The paper is now solid. I am aware that the more people read a manuscript, the more different concerns appear. I still think some minor changes would make the manuscript even better. Here are my suggestions:

**Abstract:**

***Comment 1: Lines 38-39:*** “global stress” might not be a proper term here, since the FPI measures fertility-related stress. (The same is true for Lines 456-465, 600-624 etc., where the same term is used. I think the authors may mean “total/overall fertility stress”, based on summed FPI results.)

Response 1: Thank you for your suggestion. We have changed the terminology in the manuscript from “global stress” to “fertility-related stress” throughout the manuscript.

**Background:**

***Comment 2:*** Line 65: The “severity of infertility” – at first read this seems to be referring to the medical aspects of infertility. I suggest “the severe psychological impact of infertility”.

Response 2:

Thank you for your suggestion. We have made the suggested change in our manuscript. Please see line 66-68 where it now reads *“The severe psychological impact of infertility is emphasised in a study where women with infertility reported anxiety and depression levels similar to those of women with cancer, heart attacks, and HIV (13).”*

***Comment 3:*** Lines 81-84: “discussion groups with patient partners on our research team” – This sentence is strange at this point of the paper (Introduction), where the reader is not supposed to know anything about the design (having discussion groups with patient partners). It strikes one as a quick and unexpected leap to methods and results. I suggest omitting this sentence altogether.

Response 3:

Thank you for your feedback. We agree that this sentence is not critical at this stage in the manuscript. We have omitted the following sentence: *“In discussion groups with patient partners on our research team, their lived experiences reflected these findings. Patients who did not meet their weight-loss goals reported feeling fear and avoidance toward their healthcare providers at the fertility clinic (18).”*

***Comment 4:*** Lines 89-105: The authors succeeded in giving the rationale for the methodology used. This, however, resulted in considerable redundancy in this part of the text. I suggest moving Lines 98-103 upwards (positioned after reference No 19), and shorten the yellow part (present Lines 89-98) to avoid repetitions. Also, a hypothesis for the pilot study should be clearly stated (as is present in a ‘covert form’ in Lines 533-535).

Response 4:

Thank you for your feedback. We implemented your suggestion of rearranging lines 98-103 to earlier in the paragraph and worked to make the messaging more concise. Further, we set out our hypotheses for the pilot study in this paragraph. See lines 96-98.

***Comment 5:*** Line 91: Why was the BMI cut point of 28 kg/m2 used here? Confront also with Lines 188 and 194, where the cut point seems to be 30. Please consolidate and justify.

Response 5:

Thank you for identifying this oversight. The BMI cut-off for the study was 30kg/m^2. This is now reflected in the manuscript in line 92 to align with the rest of the text.

**Methods:**

***Comment 6:*** Lines 110-117: This is a very important part emphasizing the importance of listening to the unheard voice of a stigmatized group. However, I think it belongs to the Discussion. I think Lines 108-110 are enough at this point.

Response 6:

We have made this paragraph much more succinct, leaving out further explanation of the patient-oriented research methods. The paragraph now reads: *“We utilized a dual methodological approach by combining a rapid scoping review and a pilot study to strengthen our validity of our findings while addressing unmet needs in the literature. Of note, this study used patient-oriented research methods, informing the design of the pilot study to ensure the study's objectives and methodology were meaningful to our patient population (21).”*

Further, we elected to leave the lines 110-117 out of the discussion section. We did this in an effort to keep the discussion focused and concise. We felt that our core message regarding choosing measures that were important to our population was maintained in the lines above, without being too cumbersome.

***Comment 7:*** Line 110, and further along throughout the paper: The term ‘patient partners’ may be misleading, potentially also meaning “partners of patients” (e.g. in the framework of a heteroanamnestic discussion), feasible in the infertility context, in which “the patient” is usually the couple, and not an individual. I fully understand and welcome the positive message of patients being in partnership with researchers, however, I suggest another term (e.g. “partnering patient group”, “patient focus group”, “patient collaborators”) to avoid misunderstanding.

Response 7:

Thank you for your feedback. We understand the confusion this might cause. We have reviewed our terminology, and replaced ‘patient partners’ with the term ‘people with lived experience (PWLE) collaborators’. See lines 147-149.

***Comment 8:*** Lines 143-144: “Rapid scoping review results are summarized in table format in the results section of this paper.” I suggest omitting this sentence. It is obvious that results are presented in the Results section.

Response 8:

This line has been omitted.

***Comment 9:*** Table 1: In my understanding, inclusion criteria refer to the list of requirements that all study units (subjects or, as in this case, studies) have to meet in order to qualify for the study, and exclusion criteria to what units are then decided to be omitted from the initially included pool because their inclusion would distort the results. So, exclusion criteria are not the opposite, or lack of, inclusion criteria (as in Table 1), but a further refinement or narrowing of them, applied in a second step. Therefore, in my view, the second column of Table 1 is superfluous and wrongly called exclusion. In this study, an exclusion criterion would be, for example, exercise-based lifestyle interventions combined with psychological interventions (as opposed to those including only physical exercises), or participants with BMI values over 28 who have other chronic conditions, too, which may influence the effects of exercise, etc. I suggest that the authors rethink the presentation of the inclusion/exclusion criteria.

Good! See Lines 190-192, where the exclusion process is presented in the proper way (except for age)! These (i.e. unwillingness to delay fertility treatment, physical impairments limiting ability to participate, asthma or high blood pressure as relative criteria for exclusion) are the exclusion criteria to be presented in the right column, lines 1 and 4, of Table 1!

Response 9:

Thank you for your feedback. This table has been removed and our inclusion criteria were included in text form in the preceding paragraph. See lines 125-130:
*“Articles were included in the review based on the following inclusion criteria: (1) participants were experiencing female-factor infertility, (2) participant BMIs were greater than or equal to 29kg/m^2, (3) participants were seeking fertility services, (4) interventions included an exercise component, and (5) the reported outcomes included psychosocial measures such as anxiety, depression, quality of life, fertility related stress, and hopelessness.”*

***Comment 10:*** Line 150: Please use the full term of the acronym POR on first use.

Response 10: We have replaced the abbreviation with the full term in line 150.

***Comment 11:*** Lines 164-169: The description of the questionnaires is incomplete (references, description of scales, sample items, validity and reliability data – like in Lines 393-397 regarding the HADS – are missing from here).

Response 11: Thank you for your feedback. We have elaborated on the scales used in this study in the methods section, including descriptions and validity information with relevant references. Please see lines 168-201.

***Comment 12:*** Line 207: “body weight” instead of “body weighted”.

Response 12: Thank you for your feedback. We have elected to keep our original terminology to remain consistent with the literature which we are referencing.

***Comment 13:*** Lines 209-210: I find it problematic that the exercise sessions were delivered by a kinesiologist and not a physiotherapist or a coach (trainer). This, of course, cannot be changed retrospectively, but I think this should be listed among the limitations.

Response 13:

Thank you for your feedback. The certified Kinesiologist who delivered the intervention in our study had extensive coaching experience, which we felt made her an excellent choice for the position. Further, in our view, her kinesiology background is not a limitation in the study. In Newfoundland and Labrador, Canada, where this study was conducted, Kinesiologists are considered experts in developing and implementing exercise interventions. To be a Kinesiologist, you must have an extensive educational background in exercise, movement, and biomechanics. We would argue that they would be more appropriate than a physiotherapist or personal trainer to deliver these interventions. For example, the scope of practice of physiotherapists in our region are focused on the diagnosis and treatment of injuries and physical impairments. Kinesiologists are the only professional practice to have exercise prescription in their primary scope of practice. In Canada, Kinesiologists have a four-year undergraduate degree, and thus, much more extensive training in exercise intervention development and implementation than certified personal trainers.

As a result, we have chosen not to comment on this in the limitations part of the paper. Further, where the intervention is outlined in detail in a previous publication, we have chosen not to further elaborate on the intervention in the current paper.

***Comment 14:*** Lines 220; 224-225: “The following questionnaires were administered: […] 7) Post-Intervention Semi-Structured Discussion Group” – the latter is not a questionnaire. Please reformulate.

Response 14:

This has now been addressed in lines 255-256. Following the list of questionnaires, it now reads: *“In addition, information was gathered through a post-intervention semi-structured discussion group.”*

**Results**:

***Comment 15:*** Lines 244-246: Unnecessary, can be deleted. Lines 246-250: information included in Figure 1, can be omitted, too. Figure 1: one number missing (n = ?).

Response 15:

This paragraph has been reviewed to become more concise. The paragraph now reads: *“In the initial search, 838 studies were identified through database searching and were imported to Covidence. After duplicates were removed, the remaining 662 articles were selected for title and abstract screening. Of these articles, 18 were uploaded to full-text screening for assessment of review eligibility. Ultimately, nine articles were reviewed in the rapid scoping review.”*

Figure 1 has also been reviewed to address the missing number.

***Comment 16:*** Lines 266-315: I do not find it useful for the authors to give a one-by-one, detailed description of the studies reviewed. I think they should synthesize the findings, much like they do starting from Line 316. I suggest leaving out the individual descriptions and maybe make the summary in Lines 316-324 a bit longer and more comprehensive. (Lines 318-320: Unclear sentence.)

Response 16:

Please see lines 296-332, where this summary has been reviewed to synthesize the findings of the studies reviewed. Further, lines 318-320 have been reviewed to clarify the sentence. It now reads: *“However, positive psychological effects have not been observed past immediate, end-of-study assessments, as documented in Van Dammen’s 2019 5-year post-randomization follow-up study (27).”*

***Comment 17:*** Lines 360-363: Can be omitted, has been already reported in Lines 164-169.

Response 17:
The list of surveys administered has been omitted as suggested.

***Comment 18:*** Table 4: The information on the questionnaires (such as in Lines 394-397 for the HADS; Lines 421-426 for the SF-12v2, etc.) should be included in the Methods section (Lines 164-169), not in the table legend or the Results. The Results section should only contain results.

Response 18:

Thank you for your feedback. This information has been removed from the results/table legends and has been incorporated into the Methods section. Please see lines 168-201.

***Comment 19:*** Lines 393-491: I find the reporting on the results too lengthy and detailed. Since the tables contain all the relevant information on pre-post changes, the text should, again, be a concise synthesis of the results, e.g. “Physical component scores of health-related QoL increased in the majority of the patients (4 out of 7), with the rest stagnating (1/7) or decreasing (2/7)”.

Response 19:
Thank you for your feedback. We have reviewed the results section to make the text a more concise synthesis of the data presented in the tables. For example, see lines 415-425:

*“Health-Related QoL was measured using the SF-12v2. Pre-intervention, the sample average pre-intervention for mental and physical components scores was within normal ranges. After the intervention, a general increase was observed in the physical component score to 55.7 (± 6.1) from 51.4 (± 4.3) pre-intervention. All the while, a decrease was observed in the mental component score to 32.8 (± 8.5) from 43.0 (± 8.7) pre-intervention. There was some variability in post-intervention QoL score changes amongst participants. Physical component scores increased post-intervention in four of seven participants, while one remained consistent, and two decreased. Alternatively, mental component scores decreased in four of seven participants, while one remained consistent and two increased. Of note, one participant’s mental component score decreased by over 20 points post-intervention. Table 4 describes the baseline and post-intervention Health-Related QoL scores.”*

***Comment 20:*** Lines 499-500: Here, “instructors” is used in the plural, while earlier only a single kinesiologist was mentioned in the context of intervention delivery. Please be consistent.

Response 20:

Thank you for pointing this out. This has been reviewed to reflect feedback about a single instructor terminology.

**Discussion**
***Comment 21:*** Lines 586-589: Mostly normal levels of hopelessness in the pilot study population may be attributed to the fact that the Beck Hopelessness Scale measures general and pervasive helplessness, while the patients in the discussion group may experience hopelessness focused on childbearing. This might be mentioned in the discussion.

Response 21:

This is a great point. We have included this in our discussion. Please see lines 562-568:

*“The Beck Hopelessness Scale measures general hopelessness and is not specific to fertility-related hopelessness. Therefore, our measurement tool may not have adequately captured the sentiments experienced by our PWLE collaborators. Future research should explore the role of hopelessness in people experiencing infertility, using fertility-specific hopelessness measurement tools.”*

***Comment 22:*** Lines 610-611: “Unfortunately, no normative data for the FPI tool is available, limiting the ability to compare and interpret the present study findings” – Has the FPI not been validated in the mother tongue of the participants? Elsewhere in the paper it is stated that validated questionnaires were used (e.g. in Line 668). Pilot study results could be compared to validation results either in the authors’ own culture or, in lack of a validation process, in a related culture.

Response 22: Thank you for your comment. We have reviewed this paragraph and have omitted this sentence for clarity.

***Comment 23:*** Line 648: I suggest “emerged” instead of “merged”.

Response 23: This typographical error has been addressed. Thank you for your careful and thorough review.
