# Supplementary material for: The psychosocial impact of exercise as an intervention for persons living with obesity and female infertility: A rapid scoping review and pilot study
Source: PLOS Ment Health. 2025 Dec 10;2(12):e0000202. doi: 10.1371/journal.pmen.0000202 (PMC12798248; doi:10.1371/journal.pmen.0000202)
Supplement: Attachment — Submitted filename: PLOS One - Response to Reviewer 6.docx [file pmen.0000202.s007.docx]

Reviewer #6: Given the limited input, the systematic review was reasonable as per the PRISMA guidelines. Information was varied across the studies. Previous comments appear to have been addressed adequately. They used the dual methodological approach by combining a rapid scoping review and a pilot study to strengthen the validity of their findings while addressing unmet needs in the literature. The results of both methodologies is seen in the text and tables.

**Comment 1, Reviewer 6:** Since this is a descriptive overview the ‘strength’ of the validity is not really quantitative. However, the interpretations allowed for an attempt to summarize the improvements on some domains and the ‘not’ improvements on others.

As per the authors, due to the small sample size (n=11), statistical analysis of pilot study data is limited to descriptive statistics.

This is clear from the tables presented summarizing the endpoints of interest. Confidence intervals and P-values (and perhaps even the standard deviations in some cases) for the results are not reliable due to the small sample size of the pilot study.

Many before and after statistics are presented, but this reviewer did not see many pre vs. post p-values ( even non parametrically due to the small sample size).

Why mention them and not report them and then caution against their interpretation?

Did this reviewer miss something?

The authors did much work and the effort is evident from the material presented. The conclusions followed from the observational aspects of the limited data. They did well to note the limitations of the approach throughout.

***Response to Reviewer 6:***  This manuscript has now been reviewed by six reviewers. No confidence intervals or p-values are reported in the manuscript. There are no before-and-after statistics reported. The reviewer missed something.

**“Statistical Analysis**

Due to the small sample size (n=11), statistical analysis of pilot study data is limited to descriptive statistics. For each questionnaire, individual responses before and after intervention are presented in table or figure format. A student license was obtained from QualityMetric to analyze the SF-12v2 data. PRO CoRE scoring software was used to score SF-12v2 domains and to analyze means and standard deviations of the physical and mental component scores (PCS, MCS, respectively).”
