# Supplementary material for: The psychosocial impact of exercise as an intervention for persons living with obesity and female infertility: A rapid scoping review and pilot study
Source: PLOS Ment Health. 2025 Dec 10;2(12):e0000202. doi: 10.1371/journal.pmen.0000202 (PMC12798248; doi:10.1371/journal.pmen.0000202)
Supplement: Attachment — Submitted filename: PLOS One - Response to Reviewer 6-2.docx [file pmen.0000202.s008.docx]

Reviewer #6: Given the limited input, the systematic review was reasonable as per the PRISMA guidelines. Information was varied across the studies. Previous comments appear to have been addressed adequately. They used the dual methodological approach by combining a rapid scoping review and a pilot study to strengthen the validity of their findings while addressing unmet needs in the literature. The results of both methodologies is seen in the text and tables.

**Comment 1, Reviewer 6:** Since this is a descriptive overview the ‘strength’ of the validity is not really quantitative. However, the interpretations allowed for an attempt to summarize the improvements on some domains and the ‘not’ improvements on others.

As per the authors, due to the small sample size (n=11), statistical analysis of pilot study data is limited to descriptive statistics.

This is clear from the tables presented summarizing the endpoints of interest. Confidence intervals and P-values (and perhaps even the standard deviations in some cases) for the results are not reliable due to the small sample size of the pilot study.

Many before and after statistics are presented, but this reviewer did not see many pre vs. post p-values ( even non parametrically due to the small sample size). Why mention them and not report them and then caution against their interpretation?

***Did this reviewer miss something? The authors did much work, and the effort is evident from the material presented. The conclusions followed from the observational aspects of the limited data. They did well to note the limitations of the approach throughout.*

***Response to Reviewer 6:***

We understand that the presentation of our tables and text may have given the impression that before–after inferential tests (p-values) were conducted. To clarify, no such analyses were performed.

The study was designed as a pilot with a very small sample (n=11 (pre) and n = 7 (post)), and we therefore limited the analysis to descriptive statistics only (means, standard deviations, etc.).

While we recognize the reviewer’s suggestion to include exploratory p-values (even non-parametric), we chose not to present them, as the results would be statistically misleading. Instead, we intended to highlight descriptive patterns, without implying formal significance testing.

We revised the manuscript text to more explicitly emphasize that the results are descriptive only and not based on statistical testing. We have changed any language that could imply inferential comparison between pre- and post-intervention data.

For example, the statistical analysis section now reads:

**“**Due to the small sample size (baseline n=11; post-intervention n=7), no inferential statistical testing (e.g., p-values, confidence intervals) was performed. Analyses were descriptive in nature and intended to summarize individual and group-level changes. For each questionnaire, individual responses at baseline and post-intervention are summarized in tables. For the SF-12v2, a student license was obtained from QualityMetric, and PRO CoRE scoring software was used to calculate domain scores and descriptive statistics (means and standard deviations) for the physical and mental component summaries (PCS, MCS). These results should not be interpreted as statistically significant findings, but rather as preliminary observations appropriate for a pilot study.”
